# Psychiatric disorders in professional drivers and fitness for work

**DOI:** 10.1192/j.eurpsy.2024.1193

**Published:** 2024-08-27

**Authors:** W. Ayed, G. Bahri, M. Mersni, D. Brahim, L. Houissa, I. Youssef, N. Mechergui, M. Bani, H. Bensaid, I. Yaich, C. Bensaid, N. Bram, N. Ladhari

**Affiliations:** ^1^Occupational pathology and fitness for work department Charles Nicolle hospital, Faculty of medicine of Tunis, Tunis El Manar University, Tunis; ^2^Forensic Psychiatry department, Razi Hospital, Faculty of medicine of Tunis, Tunis El Manar University, MANNOUBA, Tunisia

## Abstract

**Introduction:**

The driver’s job is a safety job requiring a meticulous neuropsychological assessment, which can affect the decision on fitness to drive. Professional driving benefits from codified regulations concerning neuropsychological disorders.

**Objectives:**

To describe the socio-professional characteristics of drivers with psychiatric illnesses

To specify the impact of these pathologies on decisions on fitness for work

**Methods:**

Retrospective descriptive study of drivers with psychiatric disorders who consulted the occupational pathology and fitness for work department of the Charles Nicolle Hospital for fitness for work assessment during the period from January 2016 to January 2023.

**Results:**

Out of 98 drivers who consulted our department for an aptitude assessment, nine (n=9) patients had a psychiatric disorder. The average age was 45±7 years. They were all men. They were bus (n=7), light car (n=1), and lorry (n=1) drivers. They belonged to the transport (n=7) and service (n=2) sectors. Length of service ranged from one year to 35 years. The pathologies presented by the patients were: anxiety-depressive disorder (n=7) , bipolar disorder (=1) and drug-addiction (n=1). They were being treated with antidepressants (n=7), anxiolytics (n=3), and thymoregulators (n=1). The medico-legal decision was to avoid professional driving (n=7) and to avoid professional driving at night (n=2).

**Conclusions:**

psychiatric illnesses can compromise fitness to work. The role of the occupational physician in the primary and secondary prevention of people at risk is important.

**Disclosure of Interest:**

None Declared

